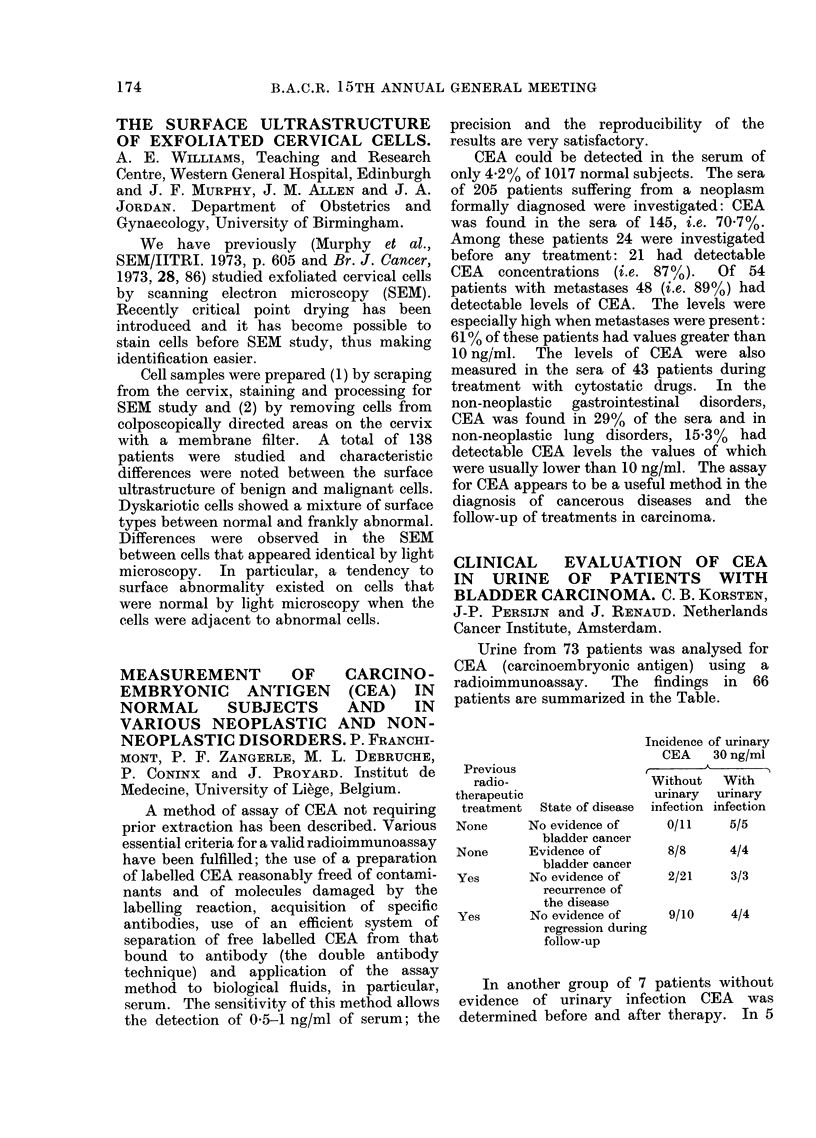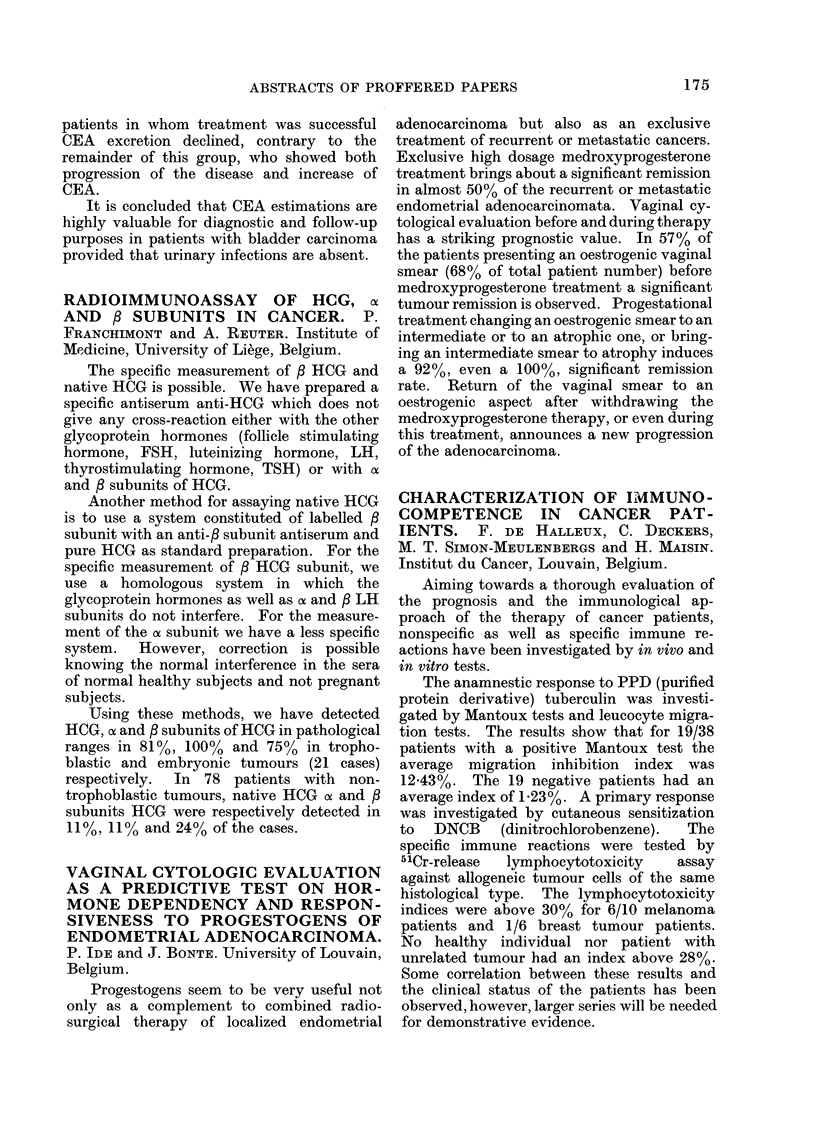# Proceedings: Clinical evaluation of CEA in urine of patients with bladder carcinoma.

**DOI:** 10.1038/bjc.1974.137

**Published:** 1974-08

**Authors:** C. B. Korsten, J. P. Persijn, J. Renaud


**CLINICAL EVALUATION OF CEA IN URINE OF PATIENTS WITH BLADDER CARCINOMA.** C. B. KORSTEN, J-P. PERSIJN and J. RENAUD. Netherlands Cancer Institute, Amsterdam.

Urine from 73 patients was analysed for CEA (carcinoembryonic antigen) using a radioimmunoassay. The findings in 66 patients are summarized in the Table.

| Previous radio-therapeutic treatment | State of disease | Incidence of urinary CEA 30 ng/ml: Without urinary infection | With urinary infection |
|---|---|---|---|
| None | No evidence of bladder cancer | 0/11 | 5/5 |
| None | Evidence of bladder cancer | 8/8 | 4/4 |
| Yes | No evidence of recurrence of the disease | 2/21 | 3/3 |
| Yes | No evidence of regression during follow-up | 9/10 | 4/4 |

In another group of 7 patients without evidence of urinary infection CEA was determined before and after therapy. In 5

patients in whom treatment was successful CEA excretion declined, contrary to the remainder of this group, who showed both progression of the disease and increase of CEA.

It is concluded that CEA estimations are highly valuable for diagnostic and follow-up purposes in patients with bladder carcinoma provided that urinary infections are absent.